# Improving the Cellular Uptake of Biomimetic Magnetic Nanoparticles

**DOI:** 10.3390/nano11030766

**Published:** 2021-03-18

**Authors:** Federica Vurro, Ylenia Jabalera, Silvia Mannucci, Giulia Glorani, Alberto Sola-Leyva, Marco Gerosa, Alessandro Romeo, Maria Grazia Romanelli, Manuela Malatesta, Laura Calderan, Guillermo R. Iglesias, María P. Carrasco-Jiménez, Concepcion Jimenez-Lopez, Massimiliano Perduca

**Affiliations:** 1 Department of Neurosciences, Biomedicine and Movement Sciences, University of Verona, 37134 Verona, Italy; federica.vurro@univr.it (F.V.); silvia.mannucci@univr.it (S.M.); marco.gerosa@univr.it (M.G.); mariagrazia.romanelli@univr.it (M.G.R.); manuela.malatesta@univr.it (M.M.); laura.calderan@univr.it (L.C.); 2 Department of Microbiology, Faculty of Sciences, University of Granada, 18071 Granada, Spain; yjabalera@ugr.es; 3 Department of Biotechnology, University of Verona, Strada Le Grazie 15, 37134 Verona, Italy; gglorani@zedat.fu-berlin.de; 4 Department of Biochemistry and Molecular Biology I, University of Granada, 18071 Granada, Spain; albertosola@ugr.es (A.S.-L.); mpazcj@ugr.es (M.P.C.-J.); 5 Instituto de Investigación Biosanitaria ibs.GRANADA, 18014 Granada, Spain; 6 Department of Computer Science, University of Verona, Strada Le Grazie 15, 37134 Verona, Italy; alessandro.romeo@univr.it; 7 Department of Applied Physic, Faculty of Sciences, University of Granada, 18071 Granada, Spain; iglesias@ugr.es

**Keywords:** biomimetic magnetic nanoparticles, poly (lactic-co-glycolic) acid, PLGA, penetrating TAT peptide, nanoparticles, magnetic hyperthermia, cellular uptake

## Abstract

*Magnetococcus marinus* magnetosome-associated protein MamC, expressed as recombinant, has been proven to mediate the formation of novel biomimetic magnetic nanoparticles (BMNPs) that are successful drug nanocarriers for targeted chemotherapy and hyperthermia agents. These BMNPs present several advantages over inorganic magnetic nanoparticles, such as larger sizes that allow the former to have larger magnetic moment per particle, and an isoelectric point at acidic pH values, which allows both the stable functionalization of BMNPs at physiological pH value and the molecule release at acidic (tumor) environments, simply based on electrostatic interactions. However, difficulties for BMNPs cell internalization still hold back the efficiency of these nanoparticles as drug nanocarriers and hyperthermia agents. In the present study we explore the enhanced BMNPs internalization following upon their encapsulation by poly (lactic-co-glycolic) acid (PLGA), a Food and Drug Administration (FDA) approved molecule. Internalization is further optimized by the functionalization of the nanoformulation with the cell-penetrating TAT peptide (TATp). Our results evidence that cells treated with the nanoformulation [TAT-PLGA(BMNPs)] show up to 80% more iron internalized (after 72 h) compared to that of cells treated with BMNPs (40%), without any significant decrease in cell viability. This nanoformulation showing optimal internalization is further characterized. In particular, the present manuscript demonstrates that neither its magnetic properties nor its performance as a hyperthermia agent are significantly altered due to the encapsulation. In vitro experiments demonstrate that, following upon the application of an alternating magnetic field on U87MG cells treated with BMNPs and TAT-PLGA(BMNPs), the cytotoxic effect of BMNPs was not affected by the TAT-PLGA enveloping. Based on that, difficulties shown in previous studies related to poor cell uptake of BMNPs can be overcome by the novel nanoassembly described here.

## 1. Introduction

Directed chemotherapy has emerged as a promising alternative to systematic treatments, allowing the selective delivery of the therapeutic agent to the target, thus reducing undesirable secondary effects. In some cases, the directed chemotherapy also allows the localized in situ combination of several therapeutic treatments [1]. In this scenario, choosing an optimal carrier becomes crucial. Within the broad range of nanocarriers so far studied, magnetic nanoparticles have become attractive candidates. On one hand, their nano scale size makes them display a larger surface area that allows them to carry relatively large amounts of the relevant molecule. On the other hand, their magnetic properties allow an external guidance and/or concentration at the target site plus the combination of therapies, such as targeted drug delivery and magnetic hyperthermia [2,3,4,5].

Although there is a wide range of magnetic nanoparticles already available on the market, they are generally produced by methods that present some drawbacks, mainly associated to the use of high temperatures, organic solvents, poor solubility in water and relatively small size (generally around 10–20 nm) [6]. Moreover, for a number of applications, including clinics, such a small size of the nanoparticles becomes a problem because it may result in a small magnetic moment per particle [7] that compromises their optimal response to an external magnetic field used for guidance/concentration or to induce magnetic hyperthermia. For clinical applications the magnetic nanoparticles should be superparamagnetic, that is, they should behave as non-magnetic in the absence of an external magnetic field (to avoid undesired nanoparticle aggregation prompted by magnetic dipole interactions), but, once a magnetic field is applied, they should respond as efficiently as possible to allow the guidance/concentration at the target [8]. Such a magnetic response depends on the magnetic moment per particle, which is strongly related to the size of superparamagnetic crystalline stoichiometric magnetite nanoparticles (MNPs) [7]. Therefore, in the context of the existing synthetic magnetic nanoparticles, an increase in their size above 20 nm (but keeping it below ~120 nm) [7] would represent a great advantage for most applications, especially related to drug delivery and magnetic hyperthermia [9].

While obtaining larger synthetic magnetic nanoparticles by using eco-friendly protocols is challenging, many of these drawbacks affecting synthetic magnetic nanoparticles are overcome in biomimetic ones (BMNPs). These are produced by taking inspiration from nature, that is, by using magnetosome membrane associated proteins, expressed as recombinant, to in vitro control the nucleation and growth process of magnetic nanoparticles synthesized from aqueous solutions. Moreover, the production of those BMNPs is eco-friendly and cost-effective [10]. MamC-mediated BMNPs have raised special interest, as they have shown in vitro to be effective drug nanocarriers and magnetic hyperthermia agents, both uncoated and coated by liposomes [4,5,11,12,13]. These BMNPs are larger than most commercial SPION and/or other biomimetic magnetites. The increased size makes them to be single magnetic domain and to have larger magnetic moment per particle than other synthetic magnetic nanoparticles or even biomimetic (Mms6-mediated) magnetic nanoparticles [10]. Moreover, BMNPs contain up to 4.5 wt% of MamC, giving them novel surface properties and providing functional groups for further functionalization [14]. In particular, it is noticeable the change in the isoelectric point (iep) of BMNPs (pH 4.4) compared to that of the inorganic magnetic nanoparticles (pH ~7.0), which allows the electrostatic bonding between the BMNPs and the relevant molecule at physiological pH values, and the spontaneous release of such a molecule at acidic pH values [14]. This is particularly interesting in the context of cancer, as it is well known the acidic environment related to tumors.

Additionally, these BMNPs have proven to be effective hyperthermia agents under the application of an alternate magnetic field (AMF) [4,5,12,14]. Temperature variations could interfere with basal metabolisms and homeostasis processes. In fact, Park et al. [15] reported that the increase of 1–2 °C from physiological temperature represents a mild heat shock for the cells, while higher temperatures lead to severe heat shock due to changes in the viscosity of membrane lipids and the transduction of a signal that induce cell heat shock responses. Cancer cells are especially sensitive to these temperature increases [16]. However, an important challenge in therapy is to combine heating effect on tumor cells avoiding damages on the surrounding healthy cells. In fact, temperatures within the range 42–46 °C result in an extremely selective thermal ablation of tumor. This effect is due to heat generation by magnetic energy dissipation from the single-domain particles caused by internal Néel fluctuations of the nanoparticle magnetic moment and external Brownian fluctuations following the application of an AMF [17]. Therefore, the use of magnetic nanoparticles able to be directed to the target and, once there, to locally increase the temperature is a promising alternative to treat cancer, being the optimum results when magnetic hyperthermia is combined with directed chemotherapy by using the magnetic nanoparticles as nanocarriers [5].

Nevertheless, to maximize the effects of the directed chemotherapy and/or magnetic hyperthermia, it is important to facilitate the cellular uptake of the BMNPs [4,5]. While placing a magnet at the target site has been shown to facilitate internalization [3,5], other strategies need to be developed to further optimize BMNPs cellular uptake. Encapsulation of BMNPs becomes an attractive candidate to improve, not only internalization, but also biocompatibility and to prevent aggregation and oxidation. Liposomes have been used to encapsulate BMNPs and such encapsulation has been proven to be effective for drug delivery [3] and to combine directed chemotherapy and magnetic hyperthermia [13]. However, such an encapsulation may not be stable when BMNPs are functionalized with drugs whose target are cell membranes, since they may disrupt the liposomes by similar mechanisms to those that disrupt/alter cell membranes. Therefore, another encapsulation strategy needs to be developed.

In this context, different natural or synthetic polymers have been used for the encapsulation of magnetic nanoparticles, such as polyethylene glycol (PEG), [18] dextran, [19] zwitterionic linear polyamidoamine, [20] hexadecantiol [21] or carbon nanomaterials [22] and cationic peptides [23,24]. However, the use of FDA-approved molecules for encapsulation, such as poly (lactic-co-glycolic) acid, commonly known as PLGA, is becoming a priority [25]. PLGA is a copolymer of the ester family, widely used to prepare nano and microparticles [26,27], when biocompatibility and biodegradability are required, such as in clinics and, specifically, for drug delivery [28,29]. PLGA has been approved for human therapies by the FDA and the European Medicines Agency (EMA) [29,30,31]. The biocompatibility of this copolymer for human cells is the result of its spontaneous hydrolysis, which leads to the release of lactic and glycolic acid monomers that can be easily metabolized by cells through the Krebs cycle.

Interestingly, encapsulations of nanoparticles that display different properties can be obtained by changing the ratio between the lactic and the glycolic acid used during the PLGA polymerization process, which allows a great versatility of the nanoformulations [32]. Moreover, due to the presence of carboxyl groups, PLGA copolymers allow further functionalization, for instance with signaling molecules that allows the target recognition and/or the internalization of the nanoformulation. Some examples are the sequence arginine-glycine-aspartate (RGD) used to recognize tumoral cells expressing integrin α_v_β_3_ on their surface [33], the apolipoprotein E modified peptide (pep-apoE), and the peptide of lipocalin-type prostaglandin-d-synthase (L-PGDS) that allow PLGA nanoparticles to cross the blood-brain barrier (BBB) [34] or the phage peptide Pep-1, which show great affinity for the interleukin 13 receptor α2 (a glioblastoma multiforme-associated plasma membrane receptor) [35]. In this scenario, TAT peptide (TATp) has been the first cationic peptide characterized as cell-penetrating peptides (CPPs) and it has been used for cancer therapy applications due to its low cytotoxicity and high tumor-penetrating ability [36,37]. Moreover, TATp-modified nanoparticles have been demonstrated to be able to cross the BBB and translocate in the nucleus of neurons in vitro and in vivo [38,39,40]. TATp is derived from the transcriptional activator protein TAT encoded by human immunodeficiency virus type 1 (HIV-1) exhibiting a positive charge in its transduction domain (RKKRRQRRR) that confers the property of rapid translocation through the plasma membrane [41]. TATp, as well as other cationic CPPs, have been widely used to deliver different cargoes, including nanoparticles into cells in vitro and in vivo [42,43,44,45].

Although this is the first study to attempt PLGA encapsulation of BMNPs, that of MNPs has already been explored by a number of authors [35,46,47]. The purpose of these studies was to produce nanocomposite that could be used as drug nanocarriers and hyperthermia agents and/or for theranostic purposes. Nevertheless, since BMNPs show differences in terms of size, magnetic properties, stability and surface properties compared to that of MNPs, as detailed above, PLGA encapsulation of BMNPs is not straightforward, and new protocols need to be set to be able to produce this novel nanocomposite.

In the context of improving PLGA(BMNPs) nanocomposites by decoration with TAT peptides, this strategy has already been used in PLGA(MNPs) nanocomposites, but there are no studies on TAT-PLGA(BMNPs). The existing studies of TAT-PLGA(MNPs) have proven efficient to bring hesperidin, naringin, and glutathione through the BBB, so effective local doses can be reached in mice brain cells bEnd.3 [48]. Developing protocols to produce novel TAT-PLGA(BMNPs) is important, since it may result in an improved efficiency of drug delivery therapies based on magnetic nanoparticles, due to the better performance, in this context, of BMNPs compared to MNPs.

However, one of the drawbacks of the encapsulation of magnetic nanoparticles is the potential shielding of the magnetic core, thus potentially compromising both the magnetic guidance of the nanoformulation as well as the ability of inducing magnetic hyperthermia. In the present study, a new nanoformulation (PLGA enveloping BMNPs) has been formulated and characterized to avoid significant shielding of the magnetic properties of the BMNPs and the ability to induce magnetic hyperthermia. How this nanoformulation increase the BMNPs cell uptake and the cytocompatibility of the nanoformulation is also investigated. The nanoformulation was further functionalized with TAT peptide to optimize cell uptake without decreasing the magnetic hyperthermia efficiency.

## 2. Materials and Methods

### 2.1. Materials

PLGA (poly[DL-lactide-co-glycolide],50:50 lactide-glycolide ratio, molecular weight 7,000–17,000, PDI 2.3, CAS 26780-50-7), ethanol (≥99% purity, CAS 64-17-5), PVA (poly[vinyl alcohol], molecular weight 30,000–70,000, and degree of alcoholysis 87–90%, CAS 9002-89-5), EDC (1-ethyl-3-(3-dimethylaminopropyl)carbodiimide, CAS 25952-53-8), NHS (N-hydroxysuccinimide, 98% purity, CAS 6066-82-6), glycine (≥99% purity, CAS 56-40-6), acetone (≥99% purity, 1.00013.) were purchased from Merck KGaA (Darmstadt, Germany). TAT peptide (Transactivator of Transcription of human immunodeficiency virus (GRKKRRQRRRPQ)) was purchased from CRIBI-Biotechnology Centre, University of Padua (Padua, Italy). Foetal bovine serum (FBS), L-glutamine, penicillin and streptomycin were obtained from Biowest (Nuaillé, France). Cell Proliferation Reagent WST-1 was acquired from Roche Diagnostic (Mannheim, Germany). *Escherichia coli* TOP10 and the plasmid pTrcHis-TOPO were purchased from Life Technologies: Invitrogen, Grand Island, NY, USA. Isopropyl-1-thio-β-D-galactopyranoside (IPTG), Na_2_CO_3_, NaHCO_3_, Fe(ClO_4_)_2_, FeCl_3_, K_4_Fe(CN)_6_, urea and HEPES were purchased from Sigma-Aldrich. Glutaraldehyde, Paraformaldehyde, OsO_4_ and Epon resin were purchased from Electron Microscopy Sciences, Hatfield, PA, USA. The U87MG cell line was purchased by ATCC Manassas, VA, USA. Eagle’s minimum essential medium (EMEM) and Dulbecco’s modified Eagle’s medium were purchased from Sigma-Aldrich. Amphotericin b (AmpB) and trypsin were purchased from Gibco, Life Technologies Inc., Grand Island, NY, USA.

### 2.2. Biomimetic Magnetic Nanoparticles Synthesis

The BMNPs used in this study were also used in a previous one [14]. Since an extensive characterization of these particles was done in this work, only basic mineral characterization is included in the present manuscript, as powder X-ray diffraction (XRD) analyses, transmission electron microscopy (TEM) and hysteresis cycle at 5K and 300K. As stated in the work above mentioned, the BMNPs used in the present study contain up to 5 wt% of MamC, show an isoelectric point of 4.4 and specific surface area of 90 m^2^/g. 

A summary of how BMNPs were synthesized is here provided. MamC expression and purification were performed as previously described by Valverde-Tercedor et al. [10]. *E. coli* TOP10 was transformed with the plasmid pTrcHis-TOPO used as a vector of the MamC protein-coding gene (Mmc1_2265) coupled to a hexahistidine tag coding sequence at its 5′ terminus. These cells were grown at 37 °C and MamC overproduction was induced by adding IPTG. Once expressed, the purification of the protein was carried out under denaturing conditions by fast protein liquid chromatography (FPLC, GE Healthcare) by using immobilized metal affinity chromatography (IMAC, GE Healthcare, Chicago, IL, USA). Lastly, dialysis was performed for a gradual removal of urea, which allowed MamC to refold progressively and the purity was evaluated by SDS-PAGE electrophoresis.

The synthesis of BMNPs was carried out at 25 °C and 1 atm total pressure from oxygen-free solutions (protocol described in Perez-Gonzalez et al. 2010 [49] and Valverde-Tercedor et al. 2015 [10]) containing 3.5 mM Na_2_CO_3_, 3.5 mM NaHCO_3_, 2.78 mM Fe(ClO_4_)_2_, 5.56 mM FeCl_3_,and 10 μg/mL recombinant MamC, at a pH value of 9. All experiments were done under anaerobic conditions inside an anaerobic Coy chamber (96% N_2_/4% H_2_, Coy Laboratory Products, Grass Lake, MI, USA). Samples were incubated for 30 days and then the solids were magnetically concentrated, washed three times with deoxygenated Milli-Q water, and stored in HEPES buffer (pH 7.4) inside the Coy Chamber at 25 °C. Samples were autoclaved before their use.

Powder X-ray diffraction analysis was carried out with an Xpert Pro X-ray diffractometer (PANalytical) using the Cu Kα radiation, 20 to 60° in 2θ (0.01°/step; 3 s per step). Precipitates were identified by XPowderX software v. 2021 (Granada, Spain) [35]. TEM analyses of the BMNPs were performed with a STEM Philips Model CM20 microscope, after embedding in Embed 812 resin and ultrathin sections (50–70 nm) preparation with a Reichert Ultracut S microtome (Leica Microsystems GmbH, Wetzlar, Germany). Crystal size was measured on 1000 nanoparticles per experiment using ImageJ 1.47.

### 2.3. PLGA Empty Nanoparticles Synthesis

The protocol used for the production of both the empty and BMNPs-embedded PLGA nanoparticles is based on the single emulsion-evaporation method [29,50] and all operations have been carried under sterile conditions The preparation of the empty NPs was the following: 10 mg of the PLGA copolymer were dissolved in 1 mL of organic solution (85% acetone and 15% ethanol). To better allow the homogenization of solvent and polymer, the preparation was sonicated for 30 s (power 8 RMS for 10 s with rest 5 s/cycle), and subsequently dripped in to 10 mL of a solution of PVA 1% in milliQ water, previously filtered with a 0.2 μM filter, maintaining a constant intensity of sonication at room temperature. The reaction mixture was stirred (2000 rpm) *overnight* at 20 °C, to evaporate the organic solvent. The preparation was pelleted at 11,000 g at 4 °C for 10′ (Eppendorf Centrifuge 5804R) and the pellet was washed three times with sterile filtered PBS solution pH 7.4. The final pellet was re-suspended in 1 mL of sterile PBS buffer and stored at 4 °C.

### 2.4. PLGA Encapsulation of BMNPs and TAT Peptide Functionalization

The encapsulation protocol for the BMNPs is based on the single emulsion-evaporation one used to prepare the empty PLGA NPs above described (Figure S1). To prevent the oxidation of the magnetic particles, all buffers and aqueous solutions were degassed under vacuum.

A total of 10 mg of the PLGA copolymer were dissolved in 1 mL of organic solution (85% acetone and 15% ethanol), together with the magnetic nanoparticles, to obtain a concentration of 0.5 mg/mL of BMNPs in the final reaction volume. The mixture was dripped in 10 mL of sterile PVA (1%) in a sonication bath, keeping the sonication intensity constant. The suspension was then left under sonication for 5′ and then kept on a flat shaker (Ika KS 260 Control—2000 rpm) at 20 °C overnight under nitrogen atmosphere to evaporate the organic phase. The preparation was pelleted using a magnet and the pellet was washed three times with sterile filtered PBS solution pH 7.4. The final pellet was re-suspended in 1 mL of sterile PBS buffer and stored at 4 °C.

To functionalize the PLGA surface of the nanoparticles with the TAT peptide, 5 mg of the previously prepared NPs were re-suspended in 1 mL of 50 mM MES, pH 5.8 and subsequently activated with 0.1 M EDC and 0.7 M NHS for 1 h a 20 °C; subsequently the NPs were recovered with a magnet and washed three times with PBS. A total of 250 μg of TAT peptide was added to the suspension and incubated overnight at 20 °C under shaking. The reaction was stopped adding glycine (20 mg/mL) and leaving the reaction at room temperature for 1 h; NPs were subsequently washed twice with PBS and the final pellet was re-suspended in 1 mL of sterile PBS buffer and stored at 4 °C.

### 2.5. Dynamic Light Scattering and Zeta Potential

Size and ζ-potential of the empty PLGA and the nanoformulations were estimated by dynamic light scattering (DLS) (Nano Zeta Sizer ZS, ZEN3600, Malvern Instruments, Malvern, Worcestershire, UK). For the size measurements, each sample was re-suspended in PBS to a final concentration between 0.1 and 1 mg/mL, being used as a stock suspension. Aliquots from this stock were withdrawn, resuspended in 10 mM NaClO_4_ and the pH was adjusted to 7.5 using 0.1M NaOH for ζ-potential measurements. All samples were measured in triplicate with the sample cell temperature fixed at 25 °C. Data were collected and analyzed by the ZetaSizer 7.10 software (Malvern, Worcestershire, UK).

### 2.6. Atomic Force Microscopy

The empty PLGA and nanoformulations were prepared for the Atomic Force Microscopy (AFM) experiments with a final concentration of 5 mg/mL. 20 μL drops of each suspension were deposited on 20 mm diameter mica discs sprayed with argon to avoid excess conglomeration of the NPs and the excess solvent was allowed to evaporate at room temperature. A NT-MDT Solver Pro microscope (Moscow, Russia), with single crystal silicon-antimony doped probe and a gold-coated tip (NSG-01 from NT-MDT) was used to collect images. The microscope was calibrated by a calibration grating (TGQ1 from NT-MDT) in order to reduce nonlinearity and hysteresis in the measurements. Scanning was performed in intermittent mode, with a frequency of 3 to 1 Hz. and the resolution of all the images acquired was 15 nm. The images were processed with the program Gwyddion and a statistical analysis over on the diameters of 30 different nanoparticles of each type was conducted to compare results to DLS data.

### 2.7. Hysteresis Cycle at 5 and 300 K and Magnetic Hyperthermia

Magnetization measurements were carried out by using a quantum design superconducting quantum interference device 5T magnetic properties measurement system (MPMS-XL SQUID magnetometer, Quantum Design, San Diego, CA, USA). Hysteresis cycles were run at 5 and 300 K.

Magnetic hyperthermia was evaluated by using a Nanotherics MagneTherm system (Warrington, UK). The alternate magnetic field apparatus is characterized by a water-cooled solenoid constituted by 17 turns and yields a magnetic field intensity of 23.133 kA/m (≈29 mT) associated with a multichannel thermometer equipped with optical fiber probes (FOTEMP4, Optocon AG, Dresden, Germany) used to assess temperature variation within the sample every 10.0 s. The nanoparticles [BMNPs, PLGA(BMNPs) and TAT-PLGA(BMNPs)] were resuspended in 1 mL water solvent with a Fe concentration of 0.5 mg/mL. Thermograms were acquired through a time window of 20 min that was chosen considering human application of the MFH and the characteristics of nanoparticles adopted. In order to maintain the samples under adiabatic conditions during the whole procedure, we set up a homemade device constituted by a thermostated closed box in which we fluxed the air stream at 37 °C.

The heat dissipation value depends on the frequency and amplitude of the applied AMF by means of two parameters, specific absorption rate (SAR) and intrinsic loss power (ILP). In particular, the transformation of magnetic energy into thermal energy mediated by magnetic nanoparticles in presence of an external AMF is quantified from the value of SAR. The SAR value of nanoparticles in solution is calculated by using the following equation:

$$\mathrm{SAR}=\frac{dT}{dt}\frac{C_{V}}{m_{Fe}},$$

where $C_{V}$ is the specific heat capacity of the solvent per g, $\frac{dT}{dt}$ is the temperature variation in time and *m_Fe_* is the mass of iron per g in the compound [51,52].

ILP was defined to evaluate the magnetothermal performance of a given suspension in order to perform a comparison between different materials without the eventual interference of the setup or the specific device used. ILP was calculated using the following equation:

$$\mathrm{ILP}=\frac{\mathrm{SAR}}{fH^{2}}.$$

For the magnetic hyperthermia characterization of the three samples, all the available frequencies (ranging from 111 to 970 kHz) of the Magnetherm device were tested. Finally, SAR and ILP were evaluated on the 111 kHz frequency, the most performant among all the possible setups and the closest to clinical applications.

### 2.8. Transmission Electron Microscopy Analysis

The U87MG cell line was cultured in EMEM with 10% (*v*/*v*) FBS, 1% (*w*/*v*) L-glutamine, 0.5% (*v*/*v*) AmpB, 100 units/mL of penicillin streptomycin (P/S), at 37 °C in a 5% CO_2_ humidified atmosphere. Cells were trypsinized when subconfluent (about 80%) and seeded on glass coverslips in 24-multiwell microplates. Cells were treated with 50 µg/mL of BMNPs, PLGA(BMNPs) and TAT-PLGA(BMNPs) for 24 h at 37 °C. In order to visualize the uptake mechanisms, cells were processed for TEM as monolayers [53]: they were fixed with 2.5% (*v*/*v*) glutaraldehyde and 2% (*v*/*v*) paraformaldehyde in 0.1 M phosphate buffer, pH 7.4, at 4 °C for 1 h, post-fixed with 1% OsO_4_ and 1.5% K_4_Fe(CN)_6_ in H_2_O for 1 h, dehydrated with acetone and embedded in Epon resin. Ultrathin sections were observed in a Philips Morgagni transmission electron microscope (FEI Company Italia Srl, Milan, Italy) operating at 80 kV and equipped with a Megaview II camera for digital image acquisition.

### 2.9. Quantitative Analysis of Nanoparticle Internalization

U87MG cells (300,000 cells per well) were seeded in 12-well plates. Then, cells were treated with BMNPs, PLGA(BMNPs) or TAT-PLGA(BMNPs) at 300 μg/mL. After 48 and 72 h of incubation, cells were washed with PBS, trypsinized, transferred to 2 mL tubes and centrifuged at 1000 rpm during 5 min. Then, 37% HCl/10% H_2_O_2_ was used to dissolve the cell pellets, and incubated at room temperature for 20 min. Subsequently 1 mL of 1% potassium thiocyanate in Milli-Q water was added and the absorbance at 490 nm was measured. A standard curve was used to obtain the endogenous iron of the cells.

### 2.10. Cell Proliferation Assay

The U87MG cell line was cultured in EMEM with 10% (*v*/*v*) FBS, 1% (*w*/*v*) Gln, 0.5% (*v*/*v*) AmpB, 100 units/mL of PS, at 37 °C in a 5% CO_2_ humidified atmosphere. Cells were trypsinized when subconfluent (about 80%) and seeded on 96-well plate for cell viability evaluation. U87MG cells were seeded (10,000 cells per well) in a 96-well plate and grown for 24 h in a humidified incubator at 37 °C with 5% CO_2_. Then, the medium was replaced with fresh medium containing different amounts of the BMNPs, PLGA, PLGA-TAT and nanoassemblies. A modified tetrazolium-based cytotoxic assay [54], the WST-1 assay was used to evaluate the cytotoxicity of the samples after 48 and 72 h. Once the time of treatment was reached, the media was replaced with 100 µL of WST solution (10%). The plate was incubated at 37 °C for 30 min and then was shaken for 10 min. Finally, the absorbance was read at a wavelength of 480 nm using a microplate reader (HTX Microplate Reader BioTek Instruments, Winooski, VT, USA). A one-way ANOVA was done with post hoc comparisons by Bonferroni test. *p* < 0.05 is considered statistically significant.

### 2.11. In Vitro Cytotoxicity of the Nanoformulation

Hyperthermia assay were performed as previously reported in [3]. Briefly, U87MG cells were seeded on 96-well plates and treated with BMNPs and the nanoassembly TAT-PLGA(BMNPs), both at a concentration of 300 μg/mL. After 48 h of incubation, cells were exposed to alternating magnetic field (frequency = 110 kHz, H = 30 kA/m) during 2 h. Finally, the viability was assessed by WST colorimetric assay, as described above. Three replicates were run to ensure reproducibility.

## 3. Results and Discussion

### 3.1. Synthesis and Characterization of Biomimetic Magnetic Nanoparticles (BMNPs) and the Nanoformulations of PLGA (BMNPs) and TAT-PLGA(BMNPs)

According to powder XRD analysis (Figure S2), MamC-mediated BMNPs were composed of >95% magnetite. TEM images revealed nanoparticles displaying well-developed crystal faces with rhombic, rectangular, and square two-dimensional morphologies (Figure S3A). Particle size distribution was 36 ± 12 nm (Figure S3B).

In order to obtain both the empty PLGA and BMNP-embedded PLGA [PLGA(BMNPs)] nanoformulations, the single emulsion-evaporation method [29,50] (Figure S1) was used. The first dimensional characterization was performed by DLS, and AFM was used to further characterize their surface (Figure S4) and dimensional properties (Table S1). Empty PLGA nanoformulations exhibit an average size close to 183 nm, both by DLS and AFM measurements, a polydispersity index (PDI) of 0.054 ± 0.007 and a ζ-potential of −3.4 ± 2.1 mV (at pH = 7.5). These nanoformulations can be easily re-suspended despite the low superficial charge and remain in suspension enough time to perform the cell experiments without significant aggregation.

PLGA nanoparticle-embedding BMNPs (PLGA(BMNPs)) exhibit larger dimensions: DLS value for the non-functionalized ones is close to 222 nm, with a PDI of 0.191 ± 0.013; the diameter is confirmed by AFM analysis (218 nm). Instead, TAT functionalized PLGA(BMNPs) (namely TAT-PLGA(BMNPs)) are even larger with a size close to 238 nm (PDI 0.194 ± 0.019) and an AFM-size of 220 nm. The lower size measured in all cases by AFM compared to that from DLS measurements is related to the techniques used: DLS measures the hydrodynamic radius, instead AFM samples have been dehydrated during the preparation to the experiment. Measurements of the ζ-potential of PLGA(BMNPs) and TAT-PLGA(BMNPs) at pH 7.5 are −15.0 ± 9.3 and −12.6 ± 5.1 mV, respectively, which is consistent with the better colloidal stability observed in the nanoformulations compared to that of the empty PLGA. The increase of the negative ζ-potential for PLGA(BMNPs), if compared to empty PLGA nanoparticles, can be attributed to the further contribution of carboxylic groups, made by the increased number of PLGA monomers participating in the nanoassemblies. This contribution is partially neutralized in the TAT-PLGA(BMNPs) by their involvement into the covalent reaction with the TAT peptide, as it is evident from the decreased negative value. All the nanoparticles analyzed by AFM exhibit a spherical shape.

### 3.2. Magnetic Saturation and Magnetic Hyperthermia of the Nanoformulations

**Figure 1:**
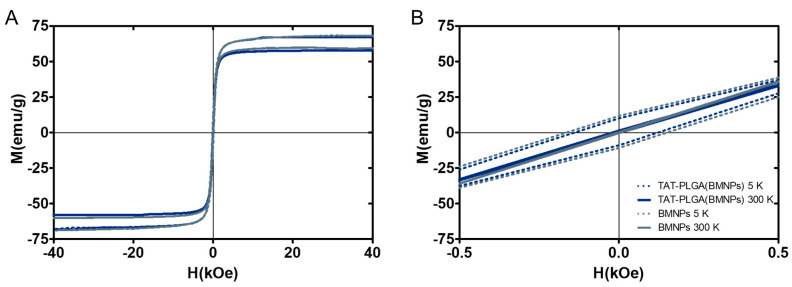
Hysteresis cycles of TAT-PLGA(BMNPs) and BMNPs. (**A**) Magnetization cycles of TAT-PLGA(BMNPs) and bare BMNPs at 5 and 300 K, showing the ferromagnetic and the superparamagnetic behavior. (**B**) Magnifications of the low-field region.

The hysteresis cycles at 5 and 300 K (Figure 1) show the ferromagnetic behavior at 5 K and the superparamagnetic behavior at 300 K [7]. At 5 K, the samples present a remnant magnetization in the absence of an external magnetic field while at 300 K, the samples showed zero magnetic coercivity. This characteristic is important, since at temperatures higher than 300 K, this sample will behave as paramagnetic (non-magnetic) in the absence of an external magnetic field, thus preventing aggregation [7,10]. However, when an external magnetic field is applied, the sample will respond efficiently, allowing magnetic guidance and/or concentration at the target site. Magnetization saturation (Ms) measurements show that the embedding of BMNPs within PLGA does not shield the magnetism of the former due to the non-magnetic coating, since the Ms for both BMNPs and TAT-PLGA(BMNPs) at 300 K is ~56 emu/g. 

**Figure 2:**
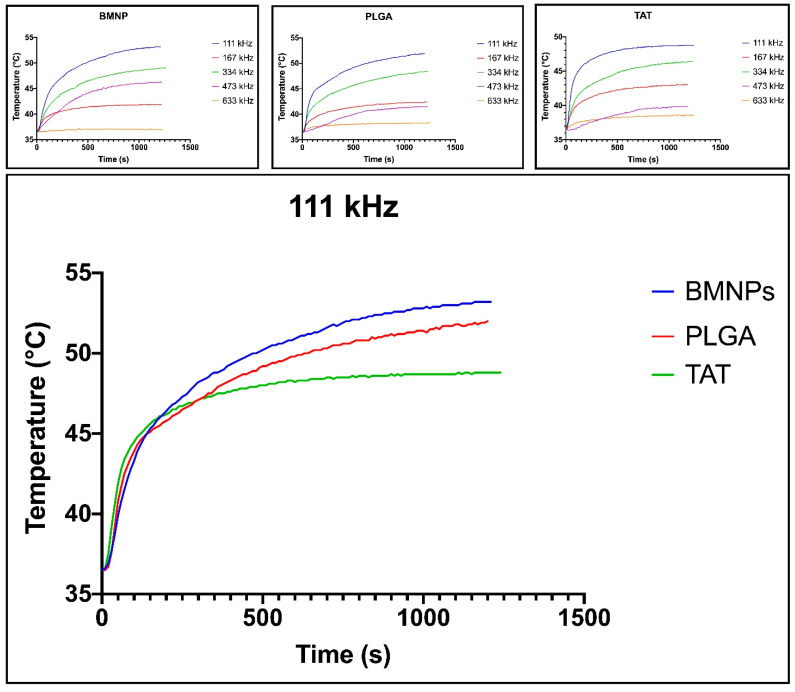
Magnetic hyperthermia data of BMNPs, PLGA(BMNPs) and TAT-PLGA(BMNPs). Hyperthermia efficiency of BMNPs, PLGA-BMNPs and TAT-PLGA-BMNPs on different frequencies (upper part of the figure) and comparison between the different nanoformulation efficiency on the 111 kHz frequency, evaluated using a Nanotherics MagneTherm system.

Magnetic hyperthermia data show that BMNPs, PLGA(BMNPs) and TAT-PLGA(BMNPs) are able to transform magnetic energy into thermal energy in presence of external AMF. Measurements of the temperature rise over time were achieved with a frequency ranging from 111 to 970 kHz were tested (Figure 2). Faster temperature rises occurred at 111 kHz. The hyperthermic efficiency is quantified by the value of SAR and ILP (Table S2). The SAR and ILP values extrapolated are comparable with them reported in literature and used in clinical trials [55].

### 3.3. Enhanced Cellular Uptake of the Nanoformulations versus BMNPs

**Figure 3:**
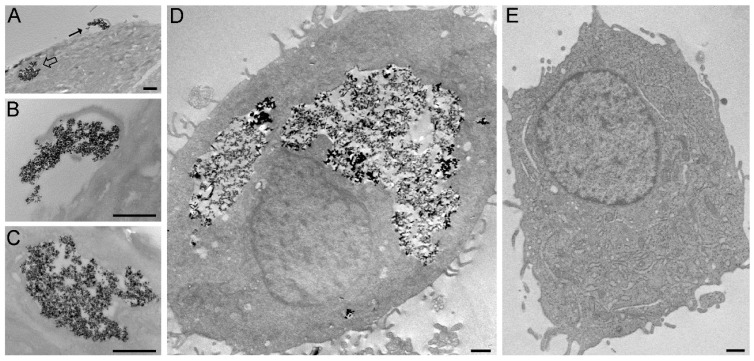
Transmission electron micrographs of cells treated for 24 h with TAT-PLGA-BMNPs (**A**–**D**) and the untreated (control) cell (**E**). (**A**) Clusters of NPs occur both at the surface (arrow) and inside (open arrow) the cell. (**B**) High magnification of the NPs at the cell surface (arrow in A): Note the cell protrusion indicating a phagocytic process. (**C**) High magnification of nanoparticles enclosed in a cytoplasmic vacuole (open arrow in **A**). (**D**) The internalized nanoparticles are stored inside large vacuoles. Bars = 1000 nm (**A**,**D**,**E**), 500 nm (**B**,**C**).

**Figure 4:**
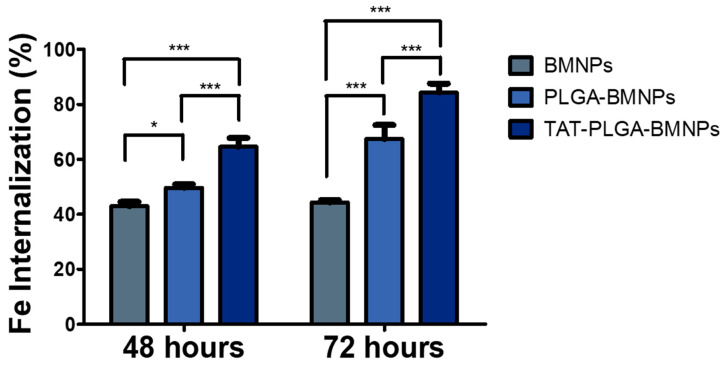
Quantitative analyses of BMNPs internalization in U87MG. Experiments were conducted twice in triplicates. Statistical differences between the treatments were considered significant when *p* values were *p* < 0.05 (*), *p* < 0.001 (***).

Ultrastructural analysis showed that BMNPs and the nanoformulations PLGA(BMNPs) and TAT-PLGA(BMNPs) enter inside the cells by endocytosis and phagocytosis, in the case of large clusters (Figure 3A,B). Once in the cytoplasm, all the BMNPs and nanoformulations are enclosed in a cytoplasmic vacuole (Figure 3D). As expected, TAT-PLGA(BMNPs) enter the cells in larger amounts in comparison to PLGA(BMNPs). This enhanced uptake is supported by data acquired by a quantitative analysis of nanoparticle internalization. Previous studies have shown that BMNPs are internalized via endocytosis when they are incubated with cells [4]. Cell internalization in the present experiments was indirectly determined by measuring the intracellular iron of the different nanoformulations [BMNPs, PLGA(BMNPs) and TAT-PLGA(BMNPs)]. As it can be seen in Figure 4, statistically significant differences were observed in iron internalized when comparing BMNPs to both PLGA(BMNPs) and TAT-PLGA(BMNPs). After 48 h, an internalization of 50% of PLGA(BMNPs) and 65% of TAT-PLGA(BMNPs) compared to 40% of BMNPs was observed. After 72 h, there is an increment in iron internalization for PLGA(BMNPs) (67%) and TAT-PLGA(BMNPs) (84%) nanoassemblies. In all cases, the internalization of TAT-PLGA(BMNPs) was always greater, up to 80% more iron internalized, demonstrating the ability of that moiety to ease or induce the internalization of the nanoformulation in the cells. In fact, TAT peptide is known as a cell-penetrating peptide and has been used to overcome the lipophilic barrier of the cellular membranes and deliver large molecules and even small particles inside the cell for biological actions [38,39,40].

### 3.4. Cytocompatibility of PLGA(BMNPs) 

**Figure 5:**
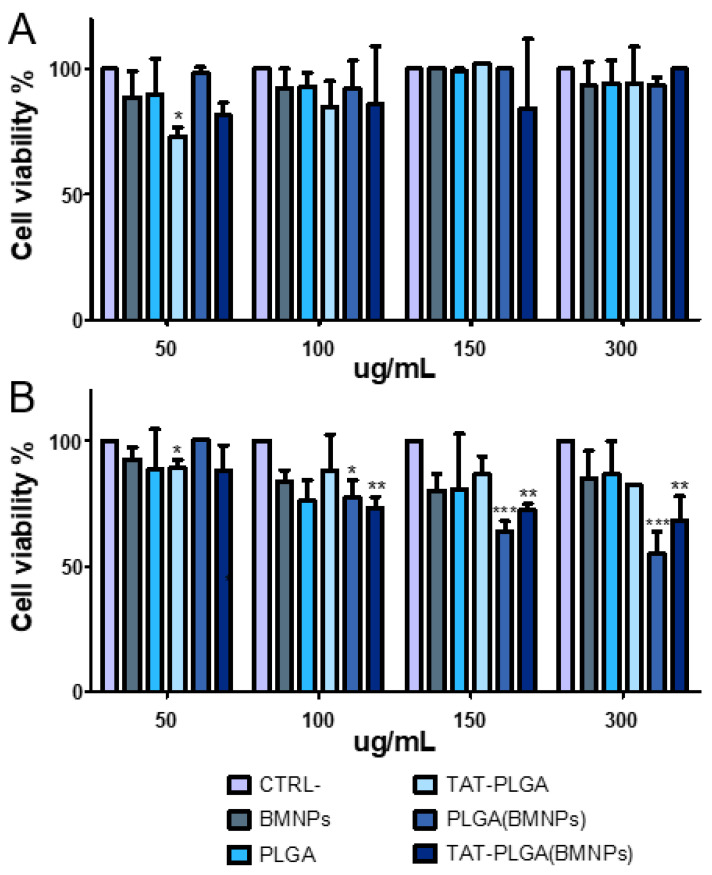
Cell proliferation assay of U87MG. U87MG cells were treated with the different samples after 48 (**A**) and 72 (**B**) hours. Statistical differences between the treatments were considered significant when *p* values were *p* ≤ 0.05 (*), *p* ≤ 0.01 (**), *p* ≤ 0.001 (***).

As shown in Figure 5A, PLGA, TAT-PLGA, BMNPs, PLGA(BMNPs) and TAT-PLGA(BMNPs) did not exert significant toxicity on cells after 48 h, demonstrating the high cytocompatibility of the samples. In fact, no sample showed significant cell viability reduction compared to control at all doses tested. These results are in agreement with previous studies that have demonstrated the cytocompatibility of nanoassemblies comprising BMNPs [4,12]. 

On the contrary, after 72 h (Figure 5B), the highest doses of PLGA(BMNPs) and TAT-PLGA(BMNPs) showed a low decrease in cell viability, >60% and >70%, respectively. This increase in cell death rate could be explained by the fact that PLGA and TAT-PLGA would promote the BMNPs internalization, compared to BMNPs control, increasing the toxicity on cells as consequence of high iron concentrations [12]. 

### 3.5. Cytotoxicity of PLGA(BMNPs) 

**Figure 6:**
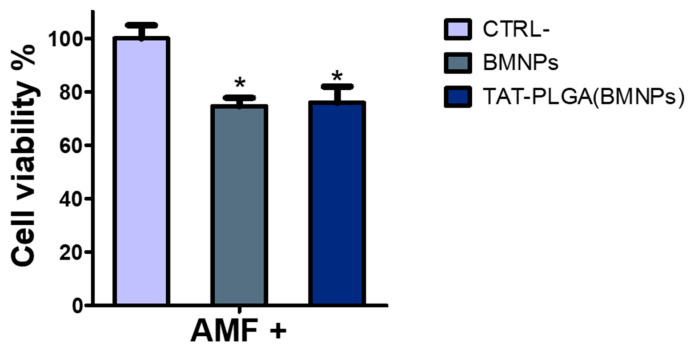
Cytotoxicity on U87MG of the nanoassembly combined with the application of an alternating magnetic field (AMF). U87MG cells were treated with BMNPs and TAT-PLGA(BMNPs) and exposed to an alternating magnetic field for 2 h. Statistical differences between the treatments were considered significant when *p* values were *p* ≤ 0.05 (*).

Results show that, under the conditions studied, both BMNPs and the nanoassembly reduced cell viability by ~30%, being such a reduction statistically significant when compared with that of the untreated cells (Figure 6). Variations in the cytotoxic effect within the two treatment were negligible. This result demonstrates that the enveloping of the BMNPs nanoparticles in PLGA, while greatly improving BMNPs uptake by cells, does not interfere with their cytotoxic effect following upon application of an alternating magnetic field. 

## 4. Conclusions

The embedding of biomimetic magnetic nanoparticles in PLGA functionalized with TAT peptide enhances the BMNPs cellular uptake with no modification of the BMNPs’ magnetic properties and/or their in vitro performance as hyperthermia agents, paving the way to the use of these nanocarriers in combined antitumoral therapy. This is the first study where a novel technology to produce PLGA-embedding BMNPs has been developed. Moreover, this technology opens the possibility of using PLGA(BMNPs) nanoformulations in different cell types by simply changing the specific targeting moiety bound to the nanoformulation surface.

## Data Availability

No new data were created or analyzed in this study. Data sharing is not applicable to this article.

## Supplementary Materials

The following are available online at https://www.mdpi.com/2079-4991/11/3/766/s1, Table S1. Size (nm) and Z-potential (mV) of PLGA nanoassemblies, empty or embedding BMNPs. Table S2. SAR and ILP values evaluated on the 111 kHz frequency. Figure S1. Representation of the PLGA(BMNPs) synthesis. Figure S2. X-ray powder diffractogram for BMNPs. Figure S3. Characterization of biomimetic magnetic nanoparticles. Figure S4. Atomic force microscopy observation of PLGA(BMNPs) and PLGA NPs.

## Abbreviations

BMNPs, biomimetic magnetic nanoparticles; MNPs, magnetite nanoparticles; PLGA, poly (lactic-co-glycolic) acid; FDA, Food and Drug Administration; TATp, TAT peptide; iep, isoelectric point; TAT-PLGA(BMNPs), TAT functionalized PLGA(BMNPs); TAT-PLGA, PLGA nanoparticles functionalized with TAT; AMF, alternate magnetic field; PEG, polyethylene glycol; EMA, European Medicines Agency; RGD, arginine-glycine-aspartate; pep-apoE, apolipoprotein E modified peptide; L-PGDS, lipocalin-type prostaglandin-d-synthase; BBB, blood–brain barrier; CPPs, cell-penetrating peptides; HIV-1, human immunodeficiency virus type 1; MNPs, magnetic nanoparticles; TAT-PLGA(MNPs), TAT functionalized PLGA containing MNPs; PVA, polyvinyl alcohol; EDC, 1-ethyl-3-(3-dimethylaminopropyl)carbodiimide; NHS, N-hydroxysuccinimide; IPTG, isopropyl-1-thio-β-D-galactopyranoside; EMEM; Eagle’s minimum essential medium; AmpB, amphotericin b; XRD; X-ray diffraction; TEM, transmission electron microscopy; FPLC, fast protein liquid chromatography; DLS, dynamic light scattering; AFM, atomic force microscopy; SAR, specific absorption rate; ILP, intrinsic loss power.
