# Supplementary material for: Improving the Cellular Uptake of Biomimetic Magnetic Nanoparticles
Source: Nanomaterials (Basel). 2021 Mar 18;11(3):766. doi: 10.3390/nano11030766 (PMC8002967; doi:10.3390/nano11030766)

Supplementary Information for

Improving the Cellular Uptake of Biomimetic Magnetic Nanoparticles

Federica Vurro [1], Ylenia Jabalera [2], Silvia Mannucci [1], Giulia Glorani [3], Alberto Sola-Leyva [4,5], Marco Gerosa [1], Alessandro Romeo [6], Maria Grazia Romanelli [1], Manuela Malatesta [1], Laura Calderan [1], Guillermo R. Iglesias [7], María P. Carrasco-Jiménez [4], Concepcion Jimenez-Lopez [2,*] and Massimiliano Perduca [3,*]

[1] Department of Neurosciences, Biomedicine and Movement Sciences, University of Verona, 37134 Verona, Italy; federica.vurro@univr.it (F.V.); silvia.mannucci@univr.it (S.M.); marco.gerosa@univr.it (M.G.); mariagrazia.romanelli@univr.it (M.G.R.); manuela.malatesta@univr.it (M.M.); laura.calderan@univr.it (L.C.)
[2] Department of Microbiology, Faculty of Sciences, University of Granada, 18071 Granada, Spain; yjabalera@ugr.es
[3] Department of Biotechnology, University of Verona, Strada Le Grazie 15, 37134 Verona, Italy; gglorani@zedat.fu-berlin.de
[4] Department of Biochemistry and Molecular Biology I, University of Granada, 18071 Granada, Spain; albertosola@ugr.es (A.S.-L.); mpazcj@ugr.es (M.P.C.-J.)
[5] Instituto de Investigación Biosanitaria ibs.GRANADA, 18014 Granada, Spain
[6] Department of Computer Science, University of Verona, Strada Le Grazie 15, 37134 Verona, Italy; alessandro.romeo@univr.it
[7] Department of Applied Physic, Faculty of Sciences, University of Granada, 18071 Granada, Spain; iglesias@ugr.es
* Correspondence: cjl@ugr.es (C.J.-L.); massimiliano.perduca@univr.it (M.P.); Tel.: +34-958249833 (C.J.-L.); +39-0458027984 (M.P.)

Number of pages: S1-S7
Number of Tables: Table S1-S2
Number of Figures: Figure S1-S4

Table S1. Size (nm) and Z-potential (mV) of PLGA nanoassemblies, empty or embedding BMNPs. DLS and AFM data diagram of empty and BMNPs embedded nanoassemblies. The Z-size, Polydispersity index (considered in intensity) and ζ-potential reported in Table 1 are the average value calculated on three measurements of three different samples; the AFM dimensions are the average between the diameters of 30 different particles considered.

Nanoassemblies	Z-size(nm)	PDI ($\times 10^{-3}$)	AFM size (nm)	ζ-pot. mV
**Empty PLGA**	**183.8** ± 55.4	**54** ± 7	**183.1** ± 53.3	**-3.4** ± 2.1
**PLGA(BMNPs)**	**221.9** ± 62.2	**191** ± 13	**218.0** ± 46.3	**-15.0** ± 9.3
**TAT-PLGA(BMNPs)**	**238.6** ± 73.2	**194** ± 19	**220.4** ± 76.3	**-12.6** ± 5.1

Table S2. SAR and ILP values evaluated on 111 kHz frequency. MFH mechanism has been evaluated on three different samples of nanoparticles: BMNPs, BMNPs coated with PLGA and PLGA coated nanoparticles functionalized with TAT peptide. The errors on dT are standard deviation based on three different measurements for each sample. The errors on SAR and ILP have been evaluated as the statistical propagation of the error from the dT standard deviation

System	Time window [s]	Frequency f [kHz]	SAR [W/g]	ILP [nHm^2kg^{-1}]	dT (°C)
**BMNPs**	100	111.0	568.5 ± 16.7	9.6 ± 0.3	6.8 ± 0.2
**PLGA(BMNPs)**	100	111.0	618.6 ± 8.4	10.0 ± 0.1	7.4 ± 0.1
**TAT-PLGA(BMNPs)**	100	111.0	668.8 ± 41.8	10.3 ± 0.7	8.1 ± 0.5

Figure S1. Representation of the PLGA(BMNPs) synthesis. A schematic representation of the preparation for both empty PLGA and BMNP embedded PLGA [PLGA(BMNPS)] nanoformulations is presented. The modified single emulsion-evaporation method is used.

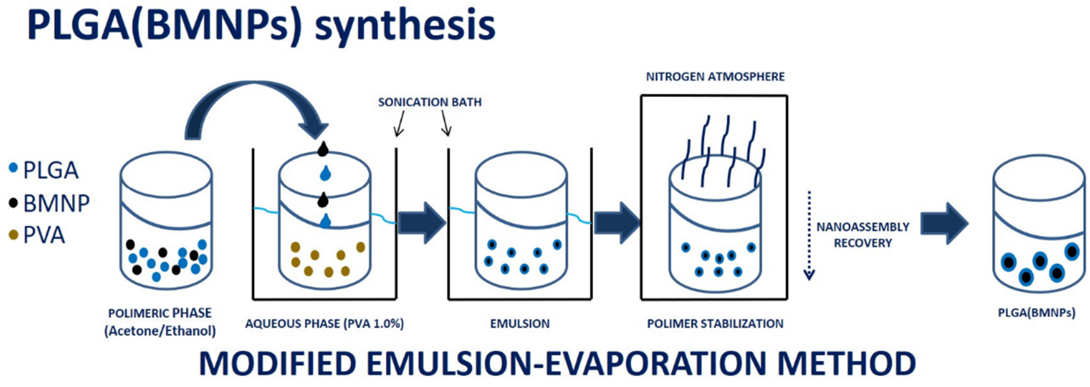

Figure S2. X-ray powder diffractogram for BMNPs. The characteristic reflections of magnetite (JCPDS file, No. 19-0629) are indicated**.**

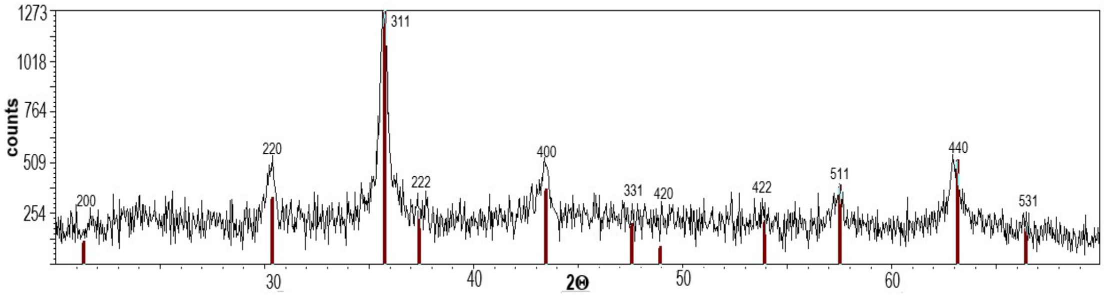

Figure S3. Characterization of biomimetic magnetic nanoparticles. TEM image (A) and diameter distribution histogram (B) of the MamC-mediated BMNPs**.** Particle size are distributed as a gaussian around the highest peak at 36 nm**.**

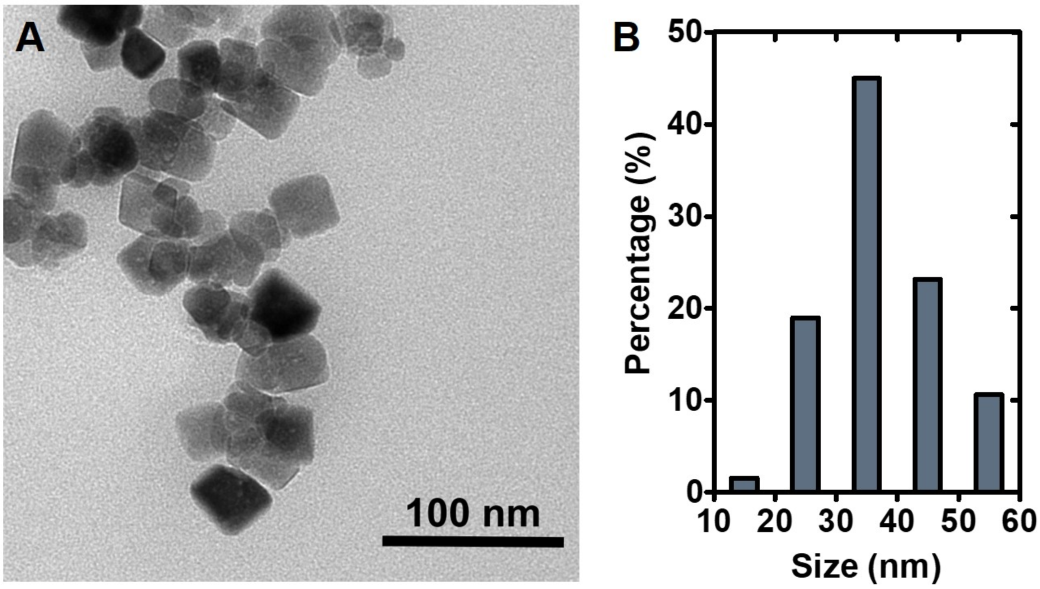

Figure S4. Atomic Force Microscopy observation of PLGA(BMNPs) and PLGA NPs. PLGA(BMNPs) visualized in intermittent mode. Magnification 7 μm × 7 μm (A) and magnification 2.5 μm × 2.5 μm (B). PLGA NPs visualized in intermittent mode. Magnification 7 μm × 7 μm (C) and magnification 2.5 μm × 2.5 μm (D). The images acquired were then used to determine the distribution of the particles size using Gwyddion software.

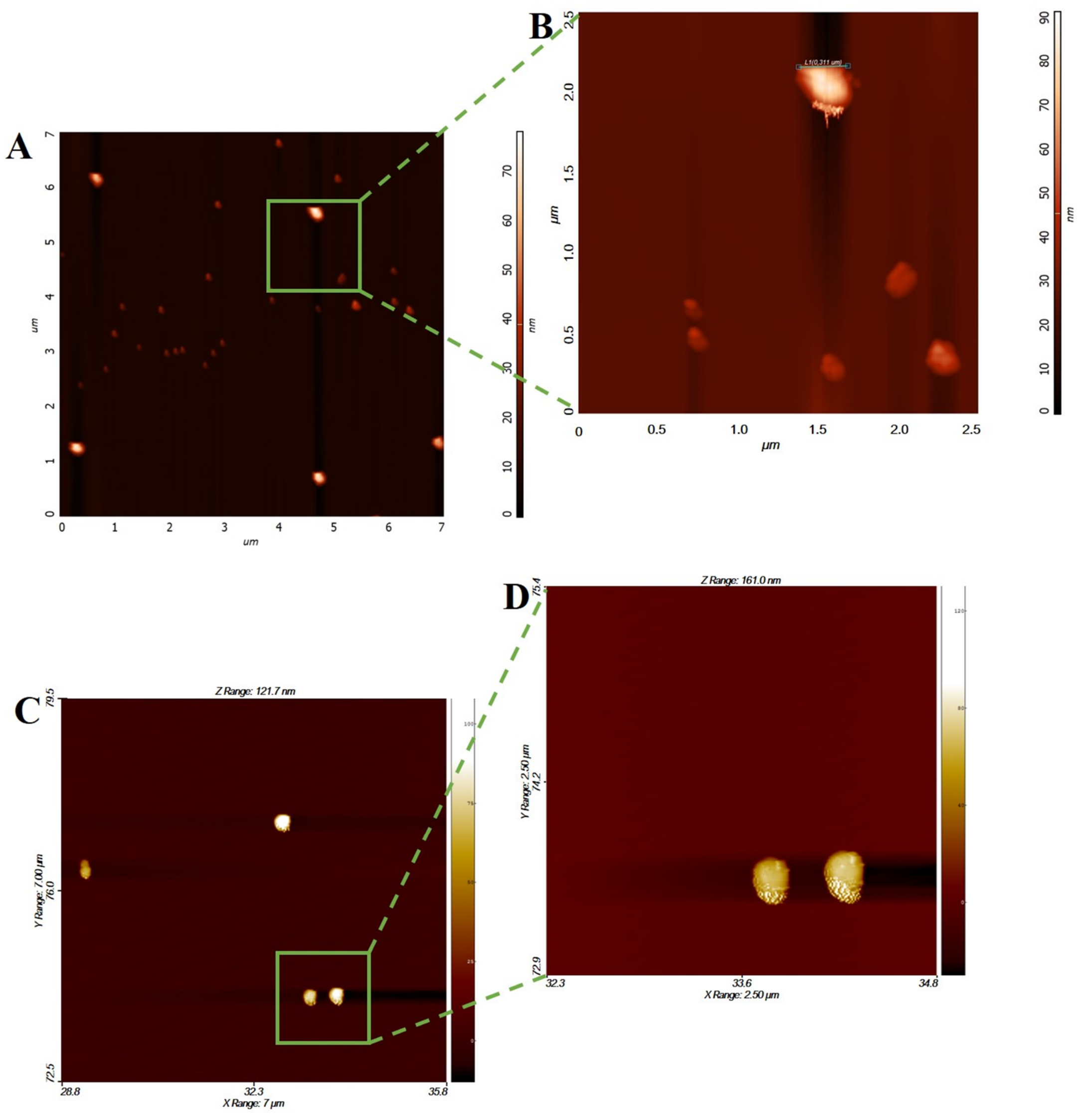

Supplement: Supplementary file 1 [file nanomaterials-11-00766-s001.pdf]
